# CellFinder: a cell data repository

**DOI:** 10.1093/nar/gkt1264

**Published:** 2013-12-03

**Authors:** Harald Stachelscheid, Stefanie Seltmann, Fritz Lekschas, Jean-Fred Fontaine, Nancy Mah, Mariana Neves, Miguel A. Andrade-Navarro, Ulf Leser, Andreas Kurtz

**Affiliations:** ^1^Berlin Brandenburg Center for Regenerative Medicine, Charité - Universitätsmedizin Berlin, Berlin 13353, Germany, ^2^Max Delbrück Center for Molecular Medicine, Computational Biology and Data Mining, Berlin 13125, Germany, ^3^Humboldt Universität zu Berlin, Institute for Computer Science, Berlin 10099, Germany and ^4^Seoul National University, College of Veterinary Medicine and Research Institute for Veterinary Science, Seoul 151-742, Republic of Korea

## Abstract

CellFinder (http://www.cellfinder.org) is a comprehensive one-stop resource for molecular data characterizing mammalian cells in different tissues and in different development stages. It is built from carefully selected data sets stemming from other curated databases and the biomedical literature. To date, CellFinder describes 3394 cell types and 50 951 cell lines. The database currently contains 3055 microscopic and anatomical images, 205 whole-genome expression profiles of 194 cell/tissue types from RNA-seq and microarrays and 553 905 protein expressions for 535 cells/tissues. Text mining of a corpus of >2000 publications followed by manual curation confirmed expression information on ∼900 proteins and genes. CellFinder’s data model is capable to seamlessly represent entities from single cells to the organ level, to incorporate mappings between homologous entities in different species and to describe processes of cell development and differentiation. Its ontological backbone currently consists of 204 741 ontology terms incorporated from 10 different ontologies unified under the novel CELDA ontology. CellFinder’s web portal allows searching, browsing and comparing the stored data, interactive construction of developmental trees and navigating the partonomic hierarchy of cells and tissues through a unique body browser designed for life scientists and clinicians.

## INTRODUCTION

The human body consists of ∼10 trillion (10^13^) individual cells (1,2). The way cells can be categorized into cell types is a source of constant debate, with published estimates on the number of different types ranging from 200 to 2260 (3,4). As ultimately any cell can be characterized by the molecules and processes it harbors at a given point in time, it is natural to base cell categorizations on the similarity of measured molecular properties of cells, like the state of the transcriptome, epigenome or proteome. Despite the great aid such a system would offer for distinguishing and analyzing development, function and dysfunction of cellular phenotypes (4,5), there is no resource available that provides a comprehensive set of measured data for different cells and cell types. This deficit is not only restricting cell biologists and medical researchers in their effort to investigate cells, but also increasingly limits scientific progress in practical applications such as the comparison between *in vitro* stem cell-derived cells and their supposed *in vivo* counterparts for regenerative medicine and in cell-based disease modeling (6).

The need for cell-focused information resources is accompanied by technological advances that facilitate the analysis of cells in even more detail, which leads to the generation of an enormous amount of cell-related data encompassing expression patterns, function, plasticity, potency, shape, intracellular structures, developmental stage and interactions with their environment. However, world wide-generated cell-, cell line- or tissue-related data are spread over a multitude of heterogeneous resources and partly only available in scientific publications, thus ‘hidden’ from conventional ways of computer-based processing. Existing integrative databases often focus on singular aspects cell-specific data. These include general gene-centered expression data repositories such as the Gene Expression Omnibus (7), Array Express (8), Gene Expression Atlas (9) and RIKEN Expression Array database (10), or specific ones, such as StemBase (11), for stem cells, or the commercial site LifeMap DiscoveryTM, which provides data on embryonic development and differentiation (12). These resources provide metadata (organisms, cell type, experimental method) at levels of detail that are highly variable between different data sets and are usually not in a standardized form. Databases and platforms such as SWISS-2D (13), MassBank (14) or the Human Protein Atlas (HPA) (15) provide complementary information on the proteome and metabolome level, respectively, sharing the same problems in terms of annotations. Data on histology, morphology and cytological parameters are available at dedicated repositories (16), galleries and educational sites (17) and some of these are partially annotated with information on the expression and location of proteins at single cell and subcellular spatial resolution (18). Only a few databases contain data at the tissue level and include developmental information, such as the mouse Gene Expression Database (GXD) (19), GUDMAP (20) or 4DXpress (21). The CELLPEDIA database classifies differentiated human cells and tissues in terms of gene expression relationships complemented by histological images (4). Still, most of the functional information on potency and development is available only in the scientific literature. Despite the multitude of resources and recent advances in providing cell-related information, no freely available platform exists that integrates the various data. Meanwhile, the potential of the growing body of biomedical knowledge and data to compare, understand, analyze, predict and synthesize cell function is rapidly increasing, especially through the recent advancements on cell-based therapies (22,23). To facilitate the exploitation of these data, CellFinder (http://www.cellfinder.org) was established to provide a one-stop portal for accessing curated information from the organ to the cell level. Therein, CellFinder provides a tool for harvesting the large scientific body of data, allows efficient data browsing and searching at the spatial (anatomical) and temporal (developmental) level together with ontology-based semantic data integration and expandability to new data types. It was designed for easy usage by life scientists and clinicians. Presently, its features are best exemplified for human kidney and liver, yet other tissues and organs are being added at constant pace. Data retrieval functions are demonstrated in a screencast at http://www.cellfinder.org/help/screencast/.

## DATA ORGANIZATION

Building the CellFinder database and portal required dedicated approaches to the organization, curation and integration of cell-related data. To organize the data in CellFinder, a novel ontology named CELDA (*Cell: Expression, Localization, Development, Anatomy*) (24) was developed. This ontology primarily provides a stable and logically sound backbone to connect existing ontologies and dictionaries covering different terms for describing cell types and lines *in vivo* and *in vitro*: Cell Ontology (CL) (25), Cell Line Ontology (CLO) (26), Experimental Factor Ontology (EFO) (27), Human Developmental Anatomy Ontology (EHDAA) (28), Foundational Model of Anatomy Ontology (FMA) (29), Adult Mouse Anatomical Dictionary (MA) (30), Gene Ontology (GO) (31) and the comparative anatomy ontology UBERON (32). In CELDA, these ontologies are linked through the top-level ontology BioTop (33) and the Basic Formal Ontology (BFO) (34). Furthermore the Relation Ontology (RO) (35) was used to standardize the relations between terms. Although CELDA is not yet part of the Open Biological and Biomedical Ontologies (OBO) foundry (36), it adheres and implements the OBO principles and mapping resources for ontology development.

By incorporating these ontologies and the supplementation with additional data not present elsewhere, CellFinder is capable of hosting the description of cell types based on species, gender, anatomical location, subcellular structures, developmental origin and molecular composition (Table 1). The conceptual backbone currently consists of 204 741 ontology terms interconnected by 5 276 442 relations and is easily expandable with further concepts from additional ontologies.

**Table 1.** Information types, data sources and content of CellFinder and CELDA

Information type	Ontologies (used in CELDA)	Data source (in addition to data from the ontology)	Current number of data entities
Cell type	CL, FMA, EFO	Manual curation	3394
Cell line	CL, CLO, EFO	hESCreg (37)	50 951
		Cellosaurus	
		Manual curation	
Cell line origin/derivation	CL, CLO	Manual curation	NA
Cells/tissue	UBERON, FMA, MA	Manual curation	535
Developmental stages	EHDAA, EFO	Manual curation	2578
Gene expression	GO	GEO (7)	205 expression profiles of 194 cell/tissue types
		Array express (8)	
		RNA-seq atlas (38)	
		StemBase (11)	
		Text mining (39,40)	
		Characterization tool (41)	
Protein expression	GO	Human protein atlas (15)	553 905 protein expressions for 535 cells/tissues
		Text mining (39,40)	
		Characterization tool (41)	
Histology/cytology	NA	Wellcome images^a^	3055 of 1790 cells/tissues
		Cell image library (16)	
		Manual curation	
Cellular components	GO, EFO	Human protein atlas (15)	3021

^a^http://wellcomeimages.org.

Not listed are data provided by individual researchers. The CELDA ontology contains currently in total 196 777 terms and 4 899 810 relations.

CL, Cell Ontology; CLO, Cell Line Ontology; EFO, Experimental Factor Ontology; EHDAA, Human Developmental Anatomy ontology; FMA, Foundational Model of Anatomy ontology; MA, Adult Mouse Anatomical Dictionary; GO, Gene Ontology; NA, not available.

The dictionary of distinct cell types used in CellFinder is thus derived from several ontologies, including those designated for cell lines (CL), anatomical (UBERON, EFO), organism (FMA, MA) and developmental information (EHDAA). However, the set of cell types, defined as phenotypically distinct cells, is still incomplete in these databases, and new cell types are continuously defined due to improved characterization methods. Therefore, we use expert knowledge to select and integrate missing tissue and developmental stage-specific cell types from literature and existing databases such as the Characterization Tool (41), hESCreg (41) and the Cellosaurus (ftp://ftp.nextprot.org/pub/current_release/controlled_vocabularies/cellosaurus.txt). CellFinder includes currently 3394 cell types distinguished by ontological terms and species (including 1058 distinct human and 489 murine cell types). A total of 1032 of these cell types have been derived through manual expert selection. Moreover, CellFinder considers 50 951 cell lines, of which 14 346 cell lines have been supplemented from literature, Cellosaurus and hESCreg, whereas the remaining is from CLO and CL.

## DATA SOURCES AND TYPES

**Figure 1.**
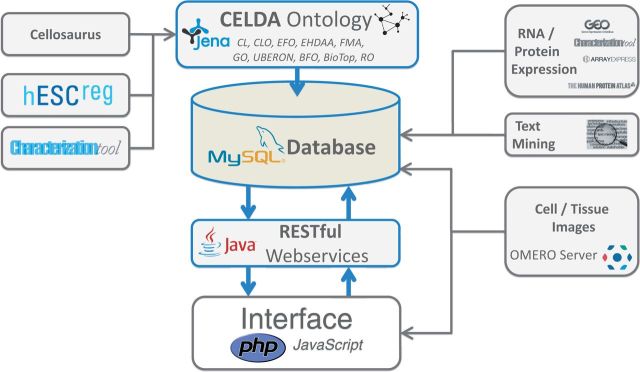
Overview of the backend, integration and access components of CellFinder. The Web site is implemented using PHP and JavaScript. The CELDA ontology is implemented with the JENA framework and translated into a MySQL database in which also the molecular and text mining data are stored. Images are stored on an OMERO server. Access to the data is provided via web services implemented in Java.

Cellular phenotypes are characterized by classical descriptors such as morphological features, shape, nucleus/cytoplasm ratio, an increasing number of intracellular components (42) and more recently by molecular descriptors such as RNA and protein expression patterns, epigenetic status and metabolic profiles. Although expression profiles complement and partially replaced the classical descriptors (4,6), they are not sufficient to fully define the phenotype of a cell; further cytological, morphological and histological images are still of high importance for describing and distinguishing cells in biology and medicine (43). Accordingly, CellFinder comprises gene and protein expression data as well as image data, both of which are integrated by the ontology-based data model (24). An overview of information currently presented in the database is given in Table 1 and Figure 1.

### RNA and protein expression data

For the exploitation of expression data, it is important to annotate analyzed samples to cell types, e.g. for the identification of reliable cell- or tissue-associated markers or gene/protein functions. Although there is an increasing amount of available gene and protein expression data available, many data sets are small scale, use specific protocols, which hinder their comparability and are often of unknown quality with respect to the cellular source. Therefore, the high-quality data sets, which were incorporated into CellFinder were selected by an expert committee of biologists and bioinformaticians, taking into account the original publications, acceptance by the community and scientific impact. Several data sets were selected to build comprehensive panels for profiling tissues and cells.

Most of the transcriptome data included in CellFinder are derived from microarrays, with the focus on tissues, normal tissue-specific cells and pluripotent stem cells, but also including some cancer cells. The transcriptome data were supplemented with RNA-seq profiles from RNA-seq Atlas (38). Protein expression data were integrated from the HPA (15). Currently CellFinder contains 205 whole genome expression profiles of 194 cell/tissue types from RNA-seq and microarrays and 553 905 protein expressions for 535 cells/tissues. A summary of the currently integrated microarray data sets and a description of the data set processing are provided in Supplementary Table S1. CellFinder is designed to allow researchers to find markers associated to particular cell types and of expression profiles for given genes or proteins, which can be useful when studying the function of a gene. Two precomputed analyses of differential expression were integrated in CellFinder: gene expression in murine samples from stem cells and derivatives from StemBase (11), and protein expression from the HPA (see Supplementary Methods for details). These analyses are accessible via the ‘Compare’ button in the CellFinder home page. In addition, the tissue-specific expression levels for genes are provided through the search option and semantic body browser (SBB) (Figure 3C and Supplementary Use Cases).

### Text mining

CellFinder also incorporates expression information derived from publications. Information extraction is carried out by a text mining pipeline followed by human expert validation. For training our machine learning algorithms and for evaluation of the methods, two corpora, each composed of 10 full-text documents were manually annotated. These documents are related to human embryonic stem cell (39) and kidney cell research, and contain a variety of entities (gene/proteins, cell types, cell lines, tissues, organs, cell components and species) and biological events (gene expression in cells and tissues and cell differentiation). Based on these gold standards, we developed a text mining pipeline for the automatic extraction of gene expression events in specific cells, cell types, cell lines or tissues (40) (Supplementary Figure S1). It is composed of the following steps: triage [using MedlineRanker (44)], preprocessing (sentence splitting, syntactic parsing), named-entity recognition, event extraction and manual validation [using Bionotate (45)]. The pipeline has been applied to >2300 full-text documents, and the derived events have been manually validated. As a result, >1800 facts on >900 distinct gene/proteins and >400 cell and tissue terms have been obtained. For integration with the other data sets, all gene names, cell types, cell lines, tissues and organs were mapped to concepts in CELDA. Evidence for extracted data is visualized using sentence-based syntax highlighting and integrated in the expression information available through the search option for cells or tissues (Supplementary Figure S2).

### Images

Imaging is an important method in cell biology to provide histological, cytological and morphological information on the cellular phenotype. A multitude of different techniques are available to generate images of cells and subcellular structural components, molecular composition and dynamics of cells and tissues. A problem for assessing and analyzing image data is the proprietary file formats that also contain the metadata defining the experimental and acquisition parameters. To store, organize and display images including their metadata in CellFinder, the Open Microscopy Environment (OMERO) (46) server was deployed. OMERO is an open-source client-server software for visualization, management and analysis of biological microscope images that supports a broad range of file formats. Data in CellFinder are also linked to anatomical images from Wikimedia (http://commons.wikimedia.org). CellFinder contains in total 3055 images of 1790 cells, tissues and organs. High-resolution microscopy images are available for 85 distinct cells and tissues.

## IMPLEMENTATION AND DATA ACCESS

The CellFinder Web site is implemented using PHP, MySQL, Java and JavaScript on a Linux server. CELDA is implemented with the JENA framework (http://jena.apache.org/) and translated into a relational database for the purpose of speed. Access to the ontological data is provided via web services. CellFinder has been carefully implemented to run on a large number of devices and different screen resolutions, although some restrictions still exist (Supplementary Table S2). The backend, integration and access components of CellFinder are summarized in Figure 1.

**Figure 2.**
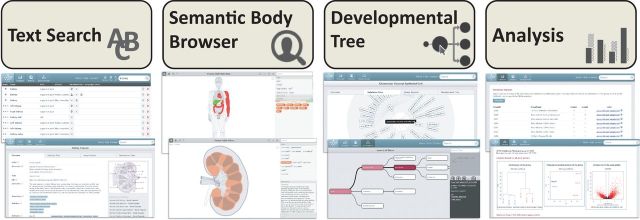
Cooperation of the different web interface components for data retrieval. Information about specific cell types can currently be retrieved by (i) direct search for organs, tissues, cell types or cell lines, (ii) browsing in the human body using the SBB and (iii) browsing the developmental tree. (iv) Comparisons of gene and protein expression data can be made in the analysis component.

Data from CellFinder are accessible from a web application via a carefully designed middleware focused on simplicity and speed to let researchers concentrate on the data rather than how to operate the application. The interface design is kept at a minimum with focus on content to allow searching, browsing and comparing the data and navigating between the hierarchical classes of cells, tissues and organs. The different web interface components for data retrieval are shown in Figure 2.

**Figure 3.**
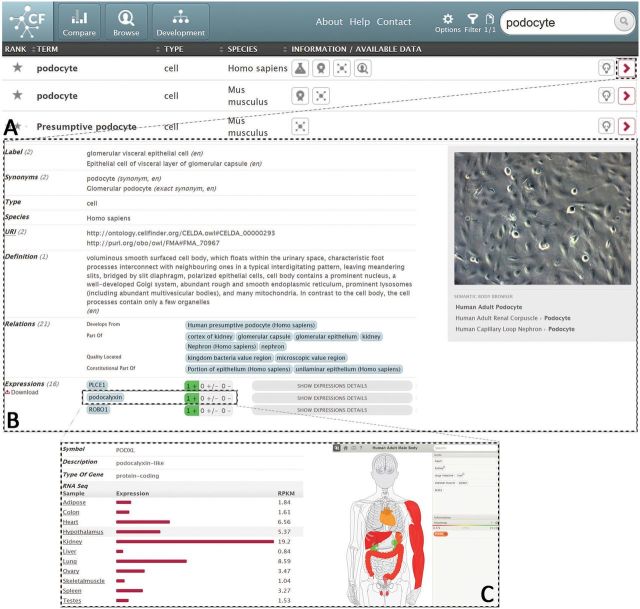
Text-based search application. The text-based search application was used to identify genes that are expressed in podocytes and compared in their expression with other tissues. (**A**) By searching for podocytes, a glomerular visceral epithelial cell of the kidney, a list of results is provided together with pictograms that identify the types of information available for each term. (**B**) Detailed information can be accessed for each term, including definition, relations and present/absent information on gene expression as well as relevant links to other databases. (**C**) Precalculated relative expression levels in different tissues (here podocalyxin, PODXL) can be displayed for each gene in the gene list as a bar plot (left) or visualized as a heatmap in the SBB (right).

CellFinder offers two general means of retrieving data via the web application, searching and browsing. Text-based searching was designed to be easy and omnipresent in CellFinder. There are no upfront extended search options, making searching as straight forward as possible. Search results are ranked according to their relevance (for details see Supplementary Methods). The results list shows basic information as well as an overview of the available data per hit. Exclusion and filtering of certain types of data from the results list is possible (Figure 3).

As biological data are complex and terms ambiguous, text searches may be time consuming and require expert knowledge. To simplify the process of information retrieval, CellFinder offers graphical tools for browsing the data. The two main graphical tools are the SBB and the Developmental Tree.

**Figure 4.**
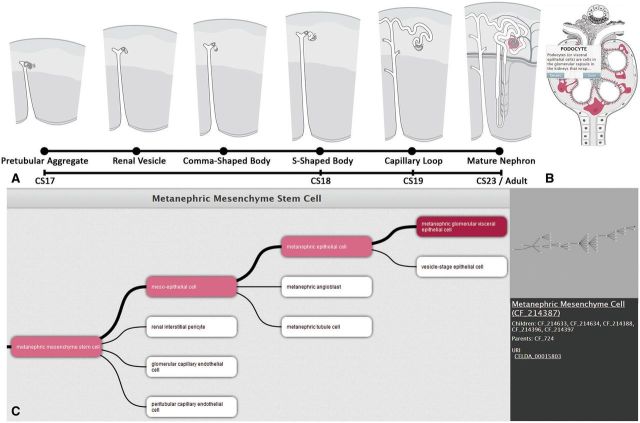
Semantic body browser (SBB) and Developmental Tree. (**A**) The SBB allows graphically explored tissues at different developmental stages (Carnegy Stage: CS) as shown for the nephron in the kidney. (**B**) The adult stage (CS23) allows zooming into the cell level by clicking on the relevant structure (red: podocytes). At all stages, relevant information is provided for the selected tissues and cells if available in CellFinder. (**C**) The Developmental Tree is linked to the cells in the SBB and provides a cell- and tissue-based hierarchical relationship tree, a section of which is shown here for the development of a metanephric glomerular visceral epithelial cell (podocyte) from a metanephric mesenchymal stem cell. The box on the upper right provides an overview of the whole tree from which the section is displayed. General information is provided for a selected cell in the tree in the lower right if available in CellFinder.

The SBB is a tool to graphically explore an organism's body and provides an entry point for browsing the CellFinder database by means of semantically annotated vector graphics. The SBB applies the ontology features to place and connect cells with the relevant tissues and organs using logical relationships (e.g. *part _of* and *has_part*) and connects the outcomes with a graphical interface (Figure 4). The Developmental Tree, on the other hand, visualizes the data as a dynamic relationship and enables the user to explore the differentiation and developmental origins and destiny of cells. The CELDA ontology supports organizing cells and tissues at different developmental states using logical connections. It automatically generates developmental trees for cells and tissues, representing ‘develops_from' or ‘develops_into' relationships and allowing developmental placement of *in vitro* and *in vivo* cell and tissue types and their associated data (Figure 4).

Moreover, the compare section was implemented to provide access to precomputed analyses of differential gene or protein expression from molecular experiments (see Supplementary Methods). In a first analysis of 241 complementary DNA microarrays from the StemBase database (11), 76 mouse samples (e.g. fibroblast, bone marrow or hematopoietic cells) were systematically compared with each other. Results of each comparison include a dendrogram, a principal component analysis and a volcano plot, and also a list of the top upregulated or downregulated gene probes. In a second analysis of data from the HPA (15), 46 human cell types were systematically compared with each other (e.g. breast glandular cells or lung macrophages). Results of each comparison consist of a list of differentially expressed proteins.

To demonstrate CellFinder data usage, use cases are provided in the Supplementary Use Cases (i) to characterize cells derived by *in vitro* stem cell differentiation by applying the SBB and (ii) to identify and characterize cell differentiation derivatives during renal differentiation with the help of the Developmental Tree.

## SUMMARY AND CONCLUSION, FUTURE DIRECTIONS

CellFinder is a data portal, which provides a unified resource of diverse data on cells. All the data available in CellFinder are of public origin and can be accessed freely using a convenient and intuitive web application.

Its implementation as an ontology-based platform allows for further expansion. For instance, we are currently working on integrating the recently developed cell phenotype ontology (CPO) (47). Furthermore the ontology allows yielding inferred relationships. These inferred relationships shall be displayed in CellFinder in the future. This includes also the ability to display more and detailed ontology and source references. Ideally any retrieved information should be easily traceable by the user. To achieve this, we are currently designing a more flexible system, which adds these references to every piece of information.

The platform is constantly expanded with further pre-analyzed data and tools for analyzing its content in a convenient manner, for instance to identify genes or protein markers that are expressed in a precise cell type and not in proximally or developmentally related ones. The need for a fully comprehensive cell type catalog and definition of classification standards is emphasized by different numbers of human cell types provided by CellFinder (1058) versus the 2260 suggested by CELLPEDIA by a combination of conventional taxonomy with physical mappings (4). To the best of our knowledge, our comprehensive and integrated view on cells is a novel and important contribution to the biomedical sciences.

CellFinder was initially focused on a few organs such as kidney and liver because of the required extensive manual data curation and data selection. Work is currently ongoing for other clinically relevant tissues, namely, the cardiovascular and hematopoietic systems. Expansion into more organs and organisms will develop through establishment of a dynamic curation process between experts and users (Supplementary Table S3). The CellFinder database has been registered at the BioDBcore catalog (http://www.biosharing.org/biodbcore) to improve its visibility to the community in support of this process.

## SUPPLEMENTARY DATA

Supplementary Data are available at NAR Online, including [48,49].

## FUNDING

Deutsche Forschungsgemeinschaft [KU 851/3-1, LE 1428/3-1 to A.K. and U.L.] and the European Commission [334502 to A.K.]. Funding for open access charge: Public Funds (Seoul National University).

*Conflict of interest statement*. None declared.
